# The Pervasiveness of Power: Dilemmas for Researchers of Major System Change in Healthcare: Comment on "‘Attending to History’ in Major System Change in Healthcare in England: Specialist Cancer Surgery Service Reconfiguration"

**DOI:** 10.34172/ijhpm.2023.7639

**Published:** 2023-08-22

**Authors:** Chris Q Smith, Iestyn Williams

**Affiliations:** School of Social Policy, University of Birmingham, Birmingham, UK

**Keywords:** Major Systems Change, Healthcare Reconfiguration, Centralisation, Power, Critical Perspectives

## Abstract

To study major system change (MSC) in healthcare, it is crucial to consider the influence of *power*. Despite this, dominant perspectives on MSC in healthcare present these as relatively neutral processes, where reconfigurations are logical solutions to clearly defined problems. Perry and colleagues’ paper adds to a growing body of research which challenges the presentation of MSC as neutral, managerial processes, instead identifying how power dynamics lie at the heart of why service change happens, how it unfolds, and its outcomes. However, the introduction of power considerations raises several overlapping methodological and ethical dilemmas for researchers, and questions regarding research design and dissemination. In this commentary, we use the insights generated by Perry et al to further explore these issues.

## Background

 Major system change (MSC) is often enacted by local providers and commissioners in England and elsewhere as a way of addressing multiple challenges in the health and social care system, including financial pressures, workforce deficits and clinical outcomes.^1^ It seems highly likely that such programmes will remain a ‘go to’ option for local policy makers, with long term operational issues combining with the effects of the COVID-19 pandemic to create huge pressures for health and social care organisations.^2^

 These initiatives are regularly presented as rational processes, informed by open consultation, to devise optimal, logical solutions to problems experienced by organisations such as acute hospitals.^3^ Despite this, there is a growing body of academic work which questions this ‘taken for granted nature of service change’^4^ (p. 1216). These critical perspectives draw attention towards crucial but neglected factors that influence the way such processes take place, including ‘local meanings, cultures and identities; leadership, co-optation and control; and framing, evidencing and strategies’^5^ (p. 1222). Out of these studies, a strong theme has emerged regarding the role of power in driving how MSCs are framed, how they unfold, and their outcomes.

 Studying the role of power in the context of MSC creates several overlapping methodological and ethical dilemmas and challenges for researchers in this area. The way Perry et al balance these dilemmas with respect to their research into MSC provides several lessons and points for consideration, which we explore in this commentary. This relates specifically to *where to direct focus *in terms of participation in the research, and whether or not the researcher should view their role as *helping *the service change proceed.

## Understanding Major System Change

 MSC in healthcare can have major implications for the wellbeing of staff,^6^ patients and the public, can swallow up substantial resources, and is often a source of conflict and disagreement between groups involved.^7^ Those seeking to study such processes must first negotiate access, invariably with those who are leading the changes. Inevitably, as with much qualitative research involving elite participants, these actors may seek to represent the official organisational position.^8,9^ Indeed, they are likely to be inclined towards a positive view of their programmes of change as evidence-based, necessary and targeted towards clinical need.^10^ However, such claims are ‘highly contested in the research community’^10^ (p. 196). For instance, in their systematic review of the methods used in economic evaluations of specialised healthcare services, Bhattarai et al find a lack of standardised evidence for the benefits of MSC. They warn this ‘may mislead decision-makers towards making wrong decisions on centralisation’^11^ (p. 1). The benefits of such changes are therefore not always as clear as sometimes presented in public plans and documents, and there may be strong competing views particularly from those groups not immediately involved in leading the change. Establishing a plurality of perspectives – particularly ones that are traditionally marginalised – can therefore be important for studies seeking to establish a ‘fully rounded’ view of MSCs and how these ‘play out’ in practice. This, in turn, promises to elucidate important power dynamics, and how these influence the ways that different groups see and act in relation to reconfigurations.

 Perry and colleagues’^12^ research provides a timely example of the benefits of establishing wide participation in MSC studies, particularly when exploring the role different understandings of history play in how groups engage in the reconfiguration. Their recruitment and data collection, taking place over a period of three years, draws testimonies from a range of professionals sitting on the various groups and committees involved in the service change. It also includes approximately 160 hours of participant observation, and analysis of 300 documents. This variety of sources and voices allows them to generate insights on conflict and disagreement within the service change, and thus more insights on the operation of power in this context. For example, the article gives significant attention to issues of framing and discursive power, particularly how ‘a tightly managed process’ (p. 2840) allowed for more contentious issues to be somewhat hidden from view. This critical analysis is enabled by the scope of the study which takes in multiple stakeholder perspectives, examining the historical deadlock between opposing professional groups and organisations involved in the change. This in turn allows for an account which goes beyond ‘simple rules’ to show how complex power dynamics involved in the process are as much a driver as any calculations and projections of the anticipated benefits.

 As noted, Perry et al add an important historical dimension to a burgeoning ethnographic literature. Ethnographic research into healthcare improvement more generally can reveal the influence of power relations and systems,^13^ and therefore helps elucidate a political dimension of MSC in healthcare that is not immediately evident otherwise. For example, in their study of the engagement carried out as part of a regional transformation plan in the National Health Service (NHS) in England, Carter and Graham explore the ‘micro-politics’ of patient and public involvement. They draw attention to how techniques such as ‘agenda framing,’ lack of transparency and ‘the exclusion of counter-narratives’ all worked to help align the activity of representation groups to the ‘radical transformation of the NHS’^14^ (p. 724). The process by which this was reached largely worked to obscure from view the ‘disparate views’ of members of the patient and public involvement group, as ‘consensus became a form of governmentality’^14^ (p. 723). Research like this again draws attention to the importance of not taking managerial accounts of the benefits of service change, and the processes involved, at face value. The presence of such disparate perspectives also brings into focus the difficulties researchers face in adjudicating between opposing accounts of the process and outcomes of MSC.

## Ethical Challenges

 Such challenges, while navigable, can pose deeper dilemmas for researchers in the stance they take towards service change and, specifically, the extent to which they should focus on generating knowledge to *help *managers implement service change. In Weberian terms,^15^ this can be framed as the extent to which academic research should seek to find solutions to issues that are practically pertinent to the *value relevance frameworks* of policy makers (the questions they see as important as defined by the ends they strive for) – or whether they exist to also critique these ends themselves. With respect to service change, there are two main standpoints a researcher might take:

1. Support – This is characteristic of the dominant ‘instrumental evaluative’ perspective outlined by Jones et al, wherein research ‘presupposes the need for change and is concerned with judging the ‘success’ of changes that have been introduced in terms of clinical or economic outcomes’^5^ (p. 1221).
2. Critique – Such perspectives can take two overlapping forms. The first entails a focus on how local policy makers strategically use knowledge to achieve their ends and make service change happen. This includes Foucauldian perspectives which scrutinise the practices of government used to legitimise large scale upheavals^16^ and the rhetorical strategies of framing used to implement change in the face of community resistance, thus undermining democratic participation.^10^ The second involves making sure views outside of dominant managerial perspectives — in terms of both staffing groups^6^ and patient and public^17^ — are given voice.

Many applied researchers adopt a somewhat ambivalent middle ground between these two standpoints. This is understandable given the difficulty of determining *prior to implementation *whether or not a change will improve outcomes, and the imperative for research to demonstrate relevance to practical policy problems.^18^ Such ambivalence is present in Perry and colleagues’^12^ paper, which at points adopts the standpoint of support, at others critique. For example, in the key messages of the article, the authors state:

 “*This study examines how, by being knowledgeable and aware of the history of previous attempts to change the way health services are provided, those organising change can make them happen”* (p. 2829).

 This statement leans towards a standpoint of ‘support’ in its concern to identify strategies that will enable successful implementation of these contested change processes. Elsewhere in the paper, Perry et al examine the ‘political nature of the changes being planned’^12^ (p. 2839). This includes the way the structure of the service change process acts as a form of ‘discursive power’ that is used to frame and channel thinking towards a course of action that diverges from the desired outcomes of some stakeholders. Of course, such observations throw into question the extent to which the outcomes of the change are themselves desirable. In this case, Perry et al note the established recommendations for reconfiguration of obstetrics and gynaecology specialist cancer surgery services as a means of reducing variation, increasing patient volumes and improving outcomes. They also note evidence for the benefits for patient outcomes of concentration of specialist services *in some contexts*. However, the disputed benefits of MSC more generally raise difficult questions for researchers regarding the extent to which their work should generate recommendations as to how managers can *make them happen. *Regrettably, there are no simple rules researchers can follow to get this right but, in our experience, they should be a constant point of reflection for those studying major systems change in healthcare. Therefore, Perry and colleagues’^12^ research successfully demonstrates the value of in-depth qualitative research into MSC in uncovering hidden power dynamics. At the same time, it also facilitates reflection on the difficult ethical challenge researchers face in how to position *themselves* to these dynamics, to which there are no easy answers.

## Ethical issues

 Not applicable.

## Competing interests

 Authors declare that they have no competing interests.
